# A Model for Mild Traumatic Brain Injury that Induces Limited Transient Memory Impairment and Increased Levels of Axon Related Serum Biomarkers

**DOI:** 10.3389/fneur.2012.00115

**Published:** 2012-07-23

**Authors:** Elham Rostami, Johan Davidsson, Kian Chye Ng, Jia Lu, Andrea Gyorgy, John Walker, Daniel Wingo, Stefan Plantman, Bo-Michael Bellander, Denes V. Agoston, Mårten Risling

**Affiliations:** ^1^Department of Neuroscience, Karolinska InstitutetStockholm, Sweden; ^2^Division of Vehicle Safety, Chalmers University of TechnologyGothenburg, Sweden; ^3^Combat Care Laboratory, Defense Medical and Environmental Research Institute, Defence Science Organisation National LaboratoriesSingapore; ^4^Department of Anatomy, Physiology and Genetics, Uniformed Services UniversityBethesda, MD, USA; ^5^Section for Neurosurgery, Department of Clinical Neuroscience, Karolinska University HospitalStockholm, Sweden

**Keywords:** mild traumatic brain injury, brain trauma model, DAI, serum biomarker, behavioral outcome, APP, S100B, NF-H

## Abstract

Mild traumatic brain injury (mTBI) is one of the most common neuronal insults and can lead to long-term disabilities. mTBI occurs when the head is exposed to a rapid acceleration-deceleration movement triggering axonal injuries. Our limited understanding of the underlying pathological changes makes it difficult to predict the outcome of mTBI. In this study we used a scalable rat model for rotational acceleration TBI, previously characterized for the threshold of axonal pathology. We have analyzed whether a TBI just above the defined threshold would induce any detectable behavioral changes and/or changes in serum biomarkers. The effect of injury on sensory motor functions, memory and anxiety were assessed by beam walking, radial arms maze and elevated plus maze at 3–7 days following TBI. The only behavioral deficits found were transient impairments in working and reference memory. Blood serum was analyzed at 1, 3, and 14 days after injury for changes in selected protein biomarkers. Serum levels of neurofilament heavy chain and Tau, as well as S100B and myelin basic protein showed significant increases in the injured animals at all time points. No signs of macroscopic injuries such as intracerebral hematomas or contusions were found. Amyloid precursor protein immunostaining indicated axonal injuries at all time points analyzed. In summary, this model mimics some of the key symptoms of mTBI, such as transient memory impairment, which is paralleled by an increase in serum biomarkers. Our findings suggest that serum biomarkers may be used to detect mTBI. The model provides a suitable foundation for further investigation of the underlying pathology of mTBI.

## Introduction

Mild or minor traumatic brain injury (mTBI) is one of the most common neurological disorders and it is estimated to comprise 70–90% of all traumatic brain injuries (Bazarian et al., 1999). Most patients recover well, however long-term problems such as headaches, vertigo, nausea, sleep disorders, depression, poor concentration, and memory problems are common (Middleboe et al., 1992; Watson et al., 1995). The mTBI patients have a brief period of unconsciousness, but the usual findings at admission to a hospital is Glasgow Coma Scale values of 13–15 and Computer tomography (CT) indicates no detectable injuries (Bazarian et al., 2006). The serum biomarker S100B has been suggested as an additional screening tool to differentiate between mTBI and more severe forms of TBI (Kovesdi et al., 2010; Unden and Romner, 2010). Currently, the understanding of mTBI and how it is diagnosed is poor. It is possible that there are a number of TBI subtypes that are categorized as mTBI. Such heterogeneity among injuries may account for the documented differences in recovery following mTBI. It is possible that the injuries resulting from some of the potential mTBI subclasses currently are not recognized in clinical practice. Because mTBI is a non-fatal injury there are few available neuropathology data from humans. There are two existing studies where the brain tissue of patients who suffered from mTBI but who subsequently died from other causes has been investigated histologically (Blumbergs et al., 1994, 1995). In both studies axonal damage was demonstrated by amyloid precursor protein (APP) staining, indicating the importance of this pathology in mTBI. Advanced neuroimaging techniques such as diffusion tensor imaging (DTI) have been used to investigate structural changes in patients with mTBI and have showed the presence of axonal injuries (Niogi et al., 2008a,b; Wilde et al., 2008; Chu et al., 2010; Mayer et al., 2010).

One of the reasons why we currently understand so little about mTBI is the lack of high fidelity, scalable models. The translation between experiments on deeply anesthetized animals and a condition, which in the clinical situation is recognized by a brief unconsciousness or occurrence of symptoms such as headache, alone is challenging. It is believed that mTBI occur when the head is exposed to loads that produce high acceleration-deceleration of the brain tissue, which in combination with its inertia induces forces in the tissue and results in diffuse injuries. One of the most frequently used models for a graded impact acceleration injury is the Marmarou’s weight drop (Marmarou et al., 1994). However, when using this model, even impact acceleration of mild severity produces hemorrhages (Folkerts et al., 1998) and concerns have been raised regarding lack of control over the precise conditions of impact (Cernak, 2005; Morales et al., 2005). Cernak et al. (2004) developed a model very similar to that of Marmarou with improved control and reproducibility of the impact to the head. Maruichi et al. (2009) constructed a model based on the methodology introduced by Cernak but with advanced ability to grade the impact. However subarachnoid and intraventricular hemorrhages in addition to hemorrhages in corpus callosum were frequently observed even at mild severity.

We have recently described a model for graded acceleration-deceleration induced TBI. We were able to identify a threshold value at which there is dispersed axonal pathology with a distribution similar to clinical diffuse axonal injuries (DAI), i.e., in the corpus callosum, but without any detectable bleeding (Davidsson and Risling, 2011; Risling et al., 2011). This acceleration-deceleration injury also results in gene expression changes in the hippocampus and an increase in serum levels of S100B. In the present study we have analyzed whether this injury results in behavioral changes or increase in serum levels of biomarkers indicative of axonal injury.

## Materials and Methods

### Experimental traumatic brain injury

**Figure 1:**
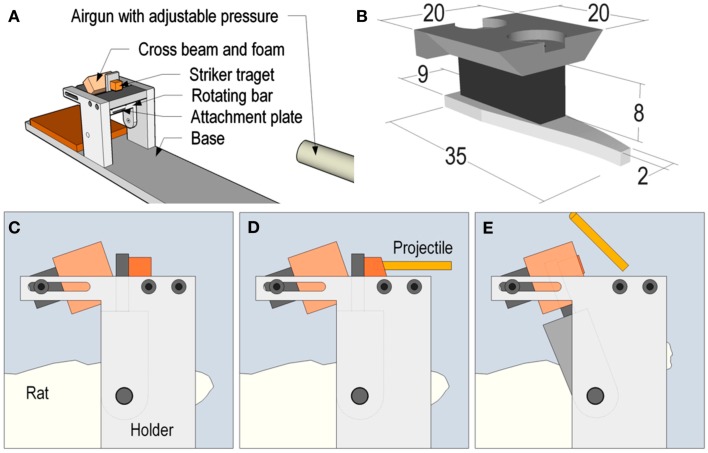
**Schematic representation of the model for rotational TBI**. In Figure **(A)**, the relation between the accelerator (modified air gun) and the head holder is represented. The attachment plate for the rat head is shown in **(B)**. A metallic level, which extends to the top of the test rig, is mounted on this attachment plate. **(C–E)** is a graphic representation of a schematic sequence which shows how the striker projectile hits the level in **(D)**, which results in the backward rotation in **(E)**.

Adult Sprague-Dawley male rats (287–474 g, *n* = 75) were used. We choose relatively larger animals since our experiments have shown better stability of the skull bone in larger animals, higher survival rate, and good repeatability. The animals were deeply anesthetized by an intra-abdominal injection of a 2.4 ml/kg mixture of 1 ml Dormicum^®^ (5 mg/ml Midazolam, Roche), 1 ml Hypnorm^®^ (Janssen) and 2 ml distilled water. Thereafter, the animals were given 0.2 ml/kg intra-muscular injections of the above mentioned mixture every 0.5 h until trauma and surgery was completed. A midline incision was made through the skin and periosteum on the skull vault, and parts of the frontal, nose, and parietal bones were freed from adherent tissue. The exposed bone was then treated with weak phosphoric acid, dried, and gently sanded prior to the fixation of a curved aluminum plate, which was shaped to match the contour of the exposed skull. The plate was attached to the bone using dental glue. Then the attachment plate was inserted and secured to a rotating bar that enabled rotation of the head in the sagittal plane. During the trauma, a solid brass weight impacted the rotating bar and the impulse produced subjected the animal heads to a short lasting sagittal plane rearward rotational acceleration, mean ± SD of the peak value were 1.5 ± 0.1 Mrad/s2, for about 0.4 ms. The sham injured control animals received identical anesthesia and surgical preparation as described above but without the trauma to the head. This trauma model has been described in detail previously (Davidsson and Risling, 2011). A schematic representation of the model for rotational TBI is presented in Figure 1. The work was performed in accordance with the Swedish National Guidelines for animal experiments, and was approved by the Animal Care and Use Ethics Committee in Stockholm.

### Behavioral testing

In the present study, we investigated the functional outcome in 11 sham operated and 12 injured animals. We used well-established models of sensory motor function (beam walking test), memory (radial arm maze; RAM), and anxiety (elevated plus maze; EPM) that have previously been used in a penetrating TBI model in our laboratory (Plantman et al., 2012).

### Beam walking test

Fine motor coordination was assessed by using a beam walking test. This test essentially examines the ability of the animal to remain upright and to walk on an elevated and relatively narrow beam (Goldstein and Davis, 1990). The setup consisted of a rigid 2.5 cm wide flat beam of 1.5 m in length leading to a brightly decorated goal-box. The beam was placed at 50 cm above the floor and foam cushions were placed underneath the beam. 3 days before habituation, animals were deprived of food until their body weight was reduced to 85% of the initial free-feeding weight (approx. 3 days). In the habituation phase (2 days) the rat was first placed into the goal-box to feed on the reward pellets provided (45 mg, Raspberry flavored, Testdiet, UK). Subsequently, progressive beam training was initiated, starting from the beam-end closest to the goal-box and progressing gradually to the opposite end. At testing, the time taken for the animals to cross the 1 m length of the beam was recorded with a stopwatch. Animals were finally tested 1 day prior to surgery. Thereafter testing was performed at 3–7 days post surgery. Animals that were unable to perform the test were designated “Immobile,” and no time was entered. However, the animals were kept and re-assessed at each testing interval.

### Elevated plus maze

Elevated plus maze relies on the animal’s preference for dark and enclosed spaces over bright, exposed areas. It involves a conflict between the desire to explore and the anxiety of exposure and height (Lau et al., 2008). The EPM has a centrally placed open platform (height above floor: 50 cm) from which four 30 cm long arms extend, two open (i.e., without walls) and two closed (i.e., with 30 cm high walls; Walf and Frye, 2007). The experimental procedure was initiated by the placement of the rat on the central platform with its head facing a closed arm. The rat was then allowed to roam the maze without any visual or audio distractions for 5 min. The whole EPM exposure was video-recorded, and various behavioral patterns were subsequently counted and timed. This included the following, (1) time spent in open arms, (2) time spent in closed arms, (3) number of rearings, and (4) number of central platform crossings. The rats were tested on the EPM at 3 days post injury.

### Radial arm maze

To measure working and reference memory, we used the RAM. The maze (Panlab, Spain) consisted of an octagonal central platform with eight automated sliding guillotine doors giving access to eight radiating arms of equal lengths (measurements of the maze is as follows: width, 1690 mm; length, 1250 mm; and height, 1450 mm). Each arm contains two pairs of photoelectrical cells mounted on the proximal and distal ends of the arm to differentiate between arm entries and visits. In addition, a food site is located at the end of each arm. The contents of the food sites are not visible from the central platform. Prominent extra-maze visual cues are present to allow spatial recognition of arm position. During habituation phase (2–3 days) the food-restricted rats were familiarized with the maze. All doors were open and food rewards were scattered around the maze to entice the rats to explore. After the rats were allowed to freely explore the maze and had consumed the food rewards at the food disk at the distal arm, the training phase started (span of 10 or more days). To measure reference memory, the rats were trained to retrieve four food pellets from each selected baited arm only once. To train the rats to do so, they were first placed in the center arena with all doors closed. After 5 s, all eight doors to the radial arms were opened and the rats were allowed to explore the RAM until the entrance into one arm was detected. Then all doors closed, except to the arm being visited. After the animal returned to the central area, the open door would close. This was followed by a confinement time (5 s) and then the doors reopened for a new round of choice. This cycle was repeated automatically until the rats visited all four baited arms or after 10 min had elapsed. For each trial, the arm choice, latency to obtain all the pellets (i.e., response latency), and the number of visits to each arm was automatically recorded by the MazeSoft software (Panlab, Spain). After each run, the maze was cleaned with absorbing paper to prevent a bias due to olfactory cues. Over time, the rats would also learn that certain arms were not baited and avoid them accordingly. Animals were trained twice each day until a stable baseline performance (>75% accuracy) was reached. After surgery and exposure to trauma or sham exposure, rats were tested daily, starting at 72 h post injury, up to 7 days.

### Reverse phase protein microarray

A total of 24 animals were used for RPPM analysis, nine exposed and nine sham operated controls equally distributed on different surviving time 1 day (*n* = 3), 3 days (*n* = 3), 14 days (*n* = 3). In addition six normal controls were included.

The animals were deeply sedated by a 2.4 ml/kg intra-abdominal injection of a mixture of 1 ml Dormicum^®^ (5 mg/ml Midazolan, Roche), 1 ml Hypnorm^®^ (Janssen), and 2 ml of distilled water and sacrificed through vast drainage of peripheral blood. The blood was drained and centrifuged and serum was collected, aliquoted, and frozen on dry ice for proteomic analysis. The primary antibodies used were for neurofilament heavy chain (NF-H; 1:20, Sigma), the microtubule associated protein total-Tau (1:20, Santa Cruz), S100B (1:20, Abcam), and myelin basic protein (MBP; 1:20, Santa Cruz). The detailed method of proteomics has been previously described (Gyorgy et al., 2010).

### Immunohistochemistry

A total of 28 animals were used for immunohistochemistry. Four injured and four sham operated controls were analyzed at 1-, 3-, and 5 days and two injured and two sham operated animals were used at 7 days.

The mid region of frozen brain tissue was cut by Cryo-Star HM 560 M (MICROM International GmbH) in coronal sections with a thickness of 14 μm and placed on Superfrost Plus slides. They were subsequently incubated in a humid chamber at 4°C for 24 h with either a rabbit polyclonal antibody against beta APP (β-APP; Zymed, dilution 1:100). Primary antibodies were diluted in a solution of 0.3% Triton, 5% bovine serum albumin, and 0.1% sodium azide in 0.01 M PBS. Donkey serum (5%) was added to minimize background staining. A Cy2-conjugated donkey anti-rabbit IgG (Jackson Immuno-Research, Inc., dilution 1:100) was used. The secondary antibodies were preabsorbed against the tissue as specified by the manufacturer. After the sections were rinsed in PBS, they were mounted in a mixture of glycerol and PBS (1:2) and cover slipped.

Sections were examined in a Nikon E600 microscope (Nikon, Shinjuku, Japan) using appropriate filter settings or a confocal C1 unit. Images were captured with a Nikon Digital Sight DS-U1 (5 megapixel) camera, controlled with Nikon NIS Elements software.

### Statistical analysis

The percent increase of each biomarker in sham and injured animals compared to normal controls were analyzed. These values were used in a two-way analysis of variances (ANOVA) with Time (1-, 3-, and 14 days) as a within-subject factor and Group (sham, injured) as a between-subjects factor. For behavioral analysis a one-way analysis of variances (ANOVA) was performed with Group (sham, injured) as a between-subjects factor. All the ANOVA analysis was followed up by pairwise comparison based on estimated marginal means and Bonferroni correction was included in all analyzes. All statistical analyses were carried out using SPSS 20.0 with an alpha level set to *p* < 0.05 (two-tailed).

## Results

### Behavioral changes

All animals survived the trauma and were behaviorally tested with beginning at 3 days following injury.

### Beam walk

Fine motor coordination was assessed by using a beam walking test. All the animals were able to remain upright and to walk on the beam at the initial trial following injury. All the animals continued to complete the test at all the following time points up to 7 days and no significant differences could be detected between the injured and the sham animals.

### Elevated plus maze

**Figure 2:**
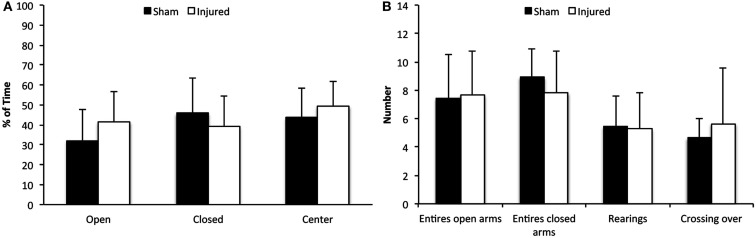
**Elevated plus maze**. Although there was a tendency of injured animals to spend more time in open arms and less time in the closed arms of the EPM, compared to control animals **(A)**, this difference was not significant. In addition, there were no significant differences in arm entries, episodes of rearing or crossings **(B)**. Data expressed as mean ± SD (*n* = 11 sham, *n* = 12 injured).

Elevated plus maze relies on the animal’s preference for dark and enclosed space over bright and exposed areas and involves a conflict between the desire to explore and the anxiety of exposure and height. The animals were tested at day 3 following injury and no significant differences were found between the shams and the injured group in time spent in closed or open arms or at the center platform (Figure 2A). Nor did we observe any significant changes in the number of total arm entries, rearing activity, or the number of crossings (Figure 2B). In conclusion, these results indicate that the animals did not experience an increase in anxiety.

### Radial arm maze

**Figure 3:**
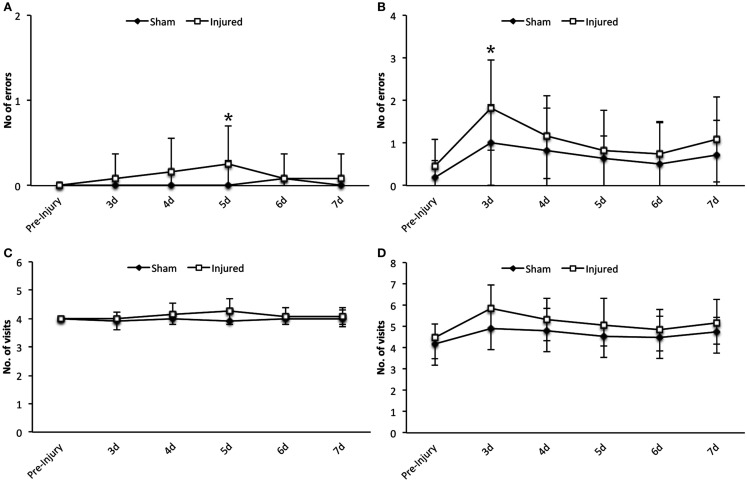
**Radial arm maze**. Statistically significant differences were detected in the number of errors in the working memory at day 5 **(A)** and in the reference memory paradigm at day 3 post injury **(B)**. However as it can be seen in the graphs the numerical difference is not vast and the sham group also show impairment in reference memory. Data expressed as mean ± SD (*n* = 11 sham, *n* = 12 injured) and significant differences are indicated by *p < 0.05. No significant differences could be observed in the number of visits in the bated arms **(C)** or all arms **(D)** between the injured and sham group (d, days).

The working and reference memory was assed by RAM from day 3 up to day 7 following injury. There was a limited but statistically significant increase in working memory errors at day 3 (*F*_1,126_ = 5.9, *p* = 0.016) and reference memory errors at day 5 (*F*_1,126_ = 5.5, *p* = 0.021) in injured animals compared to sham as illustrated in Figures 3A,B, respectively. No changes could be detected in visit response latency (time taken to correctly find all four baited arms) or the number of visits in the bated arms or all arms, at any time point following injury (Figures 3C,D).

### Changes in serum biomarker levels

**Figure 4:**
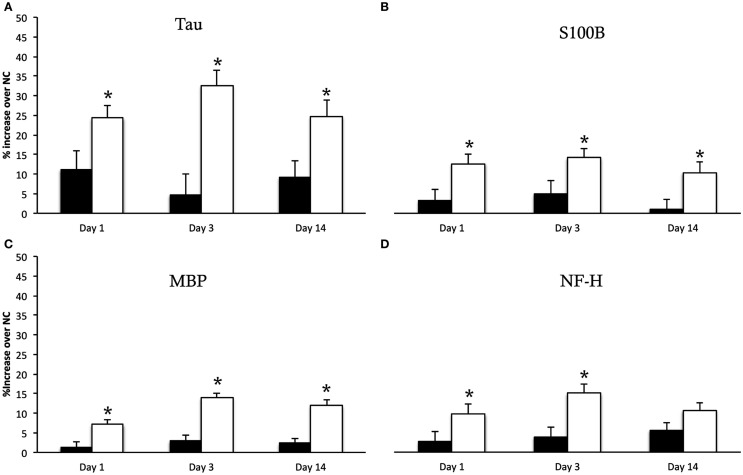
**Serum biomarkers**. *Y*-intercept values are percentage increase of each biomarker in sham animals (black bars) and injured animals (white bars) compared to normal controls (NC), given as mean ± SD. The biomarker Tau **(A)** showed the most pronounced increase following injury. The multiple comparison showed a significant increase in Tau, S100B **(B)**, MBP **(C)** at all time points compared to sham animals indicated by (*). The NF-H **(D)** showed significant increase in injured animals compared to sham at day 1 and 3 but not at 14 days following injury.

The result of RPPM values obtained in log 10 values for normal controls, sham and injured animals are given in Table 1. The percentage increase of each biomarker in both sham and injured animals compared to normal controls are illustrated in Figure 4. There was a significant difference between the sham and injured animals in all the biomarkers; S100B (*F*_1,16_ = 24.3, *p* = <0.001), MBP (*F*_1,16_ = 57.7, *p* = < 0.001), NF-H (*F*_1,16_ = 19.3, *p* = < 0.001), and Tau (*F*_1,16_ = 27.5, *p* = < 0.001). The most pronounced increase was found in Tau at all time points (Figure 4). The temporal changes were significant in MBP (*p* = 0.022) with highest values at day 3 that were sustained high at day 14. Also NF-H, S110B, and Tau showed the trend of a peak at day 3. Our follow up analysis with multiple comparisons showed a significant difference between sham and injured animals at all time points except in NF-H at day 14. The result of the follow up multiple comparisons for each biomarker is presented in Figure 4, where significant changes are indicated by (*).

**Table 1:** **Results of RPPM**.

Marker	Time	Injured	Sham
Tau (4.5 ± 0.20)	Day 1	5.66 ± 0.09	5.02 ± 0.09
	Day 3	6.10 ± 0.10	4.64 ± 0.06
	Day 14	5.72 ± 0.08	4.84 ± 0.09
MBP (5.0 ± 0.02)	Day 1	5.33 ± 0.05	5.10 ± 0.01
	Day 3	5.73 ± 0.11	5.18 ± 0.02
	Day 14	5.63 ± 0.07	5.17 ± 0.07
NF-H (5.2 ± 0.07)	Day 1	5.79 ± 0.09	5.40 ± 0.08
	Day 3	5.98 ± 0.11	5.35 ± 0.04
	Day 14	5.86 ± 0.08	5.49 ± 0.05
S100B (5.9 ± 0.28)	Day 1	6.75 ± 0.16	6.01 ± 0.04
	Day 3	7.00 ± 0.09	6.13 ± 0.02
	Day 14	6.61 ± 0.16	6.21 ± 0.12

*The results for each biomarker are given as the *Y*-intercept values obtained from the RPPM analysis. The *Y*-intercept values are given in log_10_ hence the power of each fold change is vast (*n* = 3 for each group). The values of normal controls (*n* = 6) are given in bracket. All values are given as mean ± SD*.

### Histology

No macroscopic hematomas or contusions were observed. In order to verify our previous finding of axonal injury in brain tissue in this TBI model we used β-APP. Positive β-APP immunoreactivity (IR) was found in the corpus callosum and at the border zone of the gray-white matter of the corpus callosum and the hippocampus. No β-APP-IR was found in the sham-exposed animals. These findings were in accordance with our previous reports on β-APP-IR in this TBI model (Davidsson and Risling, 2011).

## Discussion

In the present study we detected axonal injuries both by histological examination in the expected regions (Davidsson and Risling, 2011) and by significant increases of serum biomarkers following TBI. There were transient memory impairment and no other behavioral deficits could be detected. No evident intracerebral hemorrhages, contusions, or vascular lesions were found. This indicates that the acceleration impact induced in these animals produces a mTBI.

Memory deficits are the most common cognitive measure displayed in patients with minor or mild TBI (Bazarian et al., 1999; King et al., 1999; Bazarian and Atabaki, 2001; Bazarian et al., 2006). In the current study, we found transient deficits in working and reference memory but no alterations in other behavioral parameters measured. Using the same set of behavioral testing used in the current study in a penetrating TBI model, no impairment of working memory could be found (Plantman et al., 2012). However, a persistent increase of errors in reference memory was found in the injured animals. Indicating that the mild TBI in the current study do not produce permanent memory deficits while a more severe type of TBI does. Persisted behavioral deficits following severe TBI has previously been reported in other TBI models (Pierce et al., 1998). Furthermore in the penetrating TBI, we found that none of the animals could balance or move on the beam walk 3 days following injury. Although the number of animals that could move on the beam walk increased at the last day of experiment (day 7), they showed an increased latency to complete the test. In the current study no balance or sensory motor deficits were found at any time point following injury. Furthermore, the EPM showed decreased activity and level of anxiety following injury, in contrast to the current findings. In case the penetrating TBI can serve as a positive control and a more severe type of TBI, the results obtained in the present study show that our model produces mTBI. A correlation between the TBI severity and behavioral deficit have been reported in other animal TBI models (Yu et al., 2009). It should be noted that the first behavioral tests were performed 3 days following injury and there might have been more behavioral deficits in the acute phase after TBI. However, we believe that using the subacute phase rather than the acute phase, excludes possible effects of anesthesia and surgical intervention as contributing factors to behavioral impairment. This assumption is supported by the fact that the sham animals also showed a tendency of impairment at 3 days.

Previous studies have reported similar results following mTBI produced by central fluid percussion model. Using RAM Lyeth et al. (1990) demonstrated working memory deficits in absence of cell death or axonal injury in the hippocampus. Using Morris water maze in rats exposed to mild lateral fluid percussion (LFP) injury a significant impairment in memory was observed without any damaged to neurons in the hippocampus (Scheff et al., 1997). This is in line with our current and previous finding that despite the absence of structural changes in the hippocampus the animals showed transient memory impairment. Using TUNEL and Fluoro-Jade staining in present rotational TBI no evident cell death could be detected (Manuscript in preparation). Moreover, microarray analysis of the hippocampus in this TBI model showed low activity of genes related to apoptosis and cell death (Risling et al., 2011). However compared to hippocampus of animals exposed to blast- or penetrating TBI, this injury had the greatest impact on the hippocampus, measured by number of responding genes. These genes were found in functionally related groups such as cell development and differentiation, inflammatory response, receptor activity, signal transduction, and response to stress. It is possible that many of these genes are involved in recovery and regenerative processes rather than cell death mediating and deleterious effects. These issues will be the subjects of further investigation in our laboratory in addition to longer survival time for analysis of behavior outcome.

In order to get an indication of whether restricted axonal pathology can be assessed by serological biomarkers we analyzed NF-H, Tau, S100B, and MBP by RPPM. NF-H is particularly abundant in axons (Julien and Mushynski, 1998). Proteolysis of neurofilaments following TBI is believed to play an important role in axonal injury, leading to cytoskeleton disruption followed by impaired transport and swelling. The peak of serum NF-H was also found at day 3 post injury. Release of NF-H in serum has been previously studied in TBI (Anderson et al., 2008), the phosphorylated form of NF-H was analyzed and increased significantly in moderate and severe TBI but not in mTBI. Using RPPM for NF-H analysis in a blast TBI model, showed that NF-H is correlated to injury severity and that in the mild injury group it peaked 3 days after injury (Gyorgy et al., 2011). The findings in the latter study are in line with those of the present study and indicate that the axonal injury could be detected in serum up to day 3 but not later time points since no significant difference were found between sham and injured animals at day 14. An additional marker that may indicate axonal injury is Tau. In this study Tau showed the most significant percentage increase. It is a microtubule associated protein in neurons, predominantly located in axons (Binder et al., 1985). High serum levels of Tau following mTBI have been reported both in patients (Bulut et al., 2006; Kavalci et al., 2007) and in rats (Liliang et al., 2010) with conflicting results. It has been suggested by some to enable the identification of high-risk patients with mTBI (Bulut et al., 2006) while others conclude that serum Tau has limited value in this context (Kavalci et al., 2007). In the present study we detected increased serum Tau at all time points following injury, with a peak at day 3. This indicates Tau as a promising marker for mTBI. This trend was also observed in NF-H, MBP, and S100B and peaks of these biomarkers 2–3 days following injury have previously been reported. It has been shown that rats exposed to TBI produced a second peak of serum NF after 2 days (Shaw et al., 2005).

Myelin basic protein is a structural protein in the myelin sheath surrounding the axons. Myelin degradation can be expected to occur secondary to axonal injury and has been shown in brain tissue of patients with DAI (Ng et al., 1994). In animals exposed to TBI an extensive degradation of MBP was detected within hours following experimental TBI (Liu et al., 2006). In the present study high levels of MBP was detected at day 1 but most interestingly MBP peaked at day 3 and sustained high 14 days following injury. This suggests that the late MBP increase in serum is due to myelin degradation following axonal injury. This trend has also been shown in TBI patients and interpreted as myelin degradation following axonal injuries (Thomas et al., 1978; Berger et al., 2005). In addition the same trend of serum MBP, analyzed by RPPM, was shown in pigs exposed to blast TBI (Gyorgy et al., 2011). High levels of MBP were present 2 weeks following injury.

S100B is the most investigated serum biomarker in TBI (Donato, 1999). S100B has shown to correlate with TBI severity and predict normal CT-findings in mTBI (Unden and Romner, 2010). In a previous study using the present TBI model, we have shown an increase of serum S100B as early as 2–3 h following trauma, measured by ELISA (Davidsson and Risling, 2011). In this study using RPPM, we could confirm the release of S100B in serum for up to 14 days following injury. The current communication and several previous studies indicate the usefulness of RPPM as a screening tool for known biomarkers (Spurrier et al., 2008). Obviously, as in the case of all immunoassays, there is a great dependence on the specificities of antibodies used. This issue can be solved by extensive screening of antibodies by Western blotting and the use of open resources databases (Nishizuka et al., 2003). A further limitation of RPPM currently is that the protein concentration levels are relative and hence cannot be given in metric units. This can make it difficult to obtain threshold values, however the suspect biomarkers can be screened and then further investigated with methods such as ELISA for generating values to be used in the clinic.

The serum biomarkers indicated axonal injury as a result of mTBI during this study which corresponds to current and previous histological findings of axonal lesions by using β-APP (Davidsson and Risling, 2011). The significance of axonal injury in mTBI has been previously demonstrated in patients (Blumbergs et al., 1994, 1995).

In summary, the transient memory impairment in combination with the absence of other behavioral deficits in this model indicates that the TBI produced corresponds to a mild type of TBI. Furthermore, the main histological findings using this model were axonal injuries. Interestingly, this could also be detected by biomarkers in serum. This suggests that this model can be used for further analysis of underlying pathology causing mTBI as well as further investigation of possible biomarkers that can be used in clinical settings as diagnostic and prognostic tools.

## Conflict of Interest Statement

The authors declare that the research was conducted in the absence of any commercial or financial relationships that could be construed as a potential conflict of interest.
